# Molecular characterization revealed the role of thaumatin-like proteins of *Rhizoctonia solani* AG4-JY in inducing maize disease resistance

**DOI:** 10.3389/fmicb.2024.1377726

**Published:** 2024-05-15

**Authors:** Jiayue Liu, Shang Feng, Tingting Liu, Yanan Mao, Shen Shen, Yuwei Liu, Zhimin Hao, Zhiyong Li

**Affiliations:** ^1^Institute of Millet Crops, Hebei Academy of Agriculture and Forestry Sciences/Key Laboratory of Genetic Improvement and Utilization for Featured Coarse Cereals (Co-construction by Ministry and Province), Ministry of Agriculture and Rural Affairs/The Key Research Laboratory of Minor Cereal Crops of Hebei Province, Shijiazhuang, China; ^2^State Key Laboratory of North China Crop Improvement and Regulation/Hebei Bioinformatic Utilization and Technological Innovation Center for Agricultural Microbes, Hebei Agricultural University, Baoding, China

**Keywords:** thaumatin-like proteins, secreted protein, elicitor, *Rhizoctonia solani*, AG4-JY

## Abstract

The gene family of thaumatin-like proteins (TLPs) plays a crucial role in the adaptation of organisms to environmental stresses. In recent years, fungal secreted proteins (SP) with inducing disease resistance activity in plants have emerged as important elicitors in the control of fungal diseases. Identifying SPs with inducing disease resistance activity and studying their mechanisms are crucial for controlling sheath blight. In the present study, 10 proteins containing the thaumatin-like domain were identified in strain AG4-JY of *Rhizoctonia solani* and eight of the 10 proteins had signal peptides. Analysis of the TLP genes of the 10 different anastomosis groups (AGs) showed that the evolutionary relationship of the TLP gene was consistent with that between different AGs of *R. solani*. Furthermore, it was found that RsTLP3, RsTLP9 and RsTLP10 were regarded as secreted proteins for their signaling peptides exhibited secretory activity. Prokaryotic expression and enzyme activity analysis revealed that the three secreted proteins possess glycoside hydrolase activity, suggesting they belong to the TLP family. Additionally, spraying the crude enzyme solution of the three TLP proteins could enhance maize resistance to sheath blight. Further analysis showed that genes associated with the salicylic acid and ethylene pathways were up-regulated following RsTLP3 application. The results indicated that RsTLP3 had a good application prospect in biological control.

## Introduction

*Rhizoctonia solani* is the pathogen responsible for sheath blight. It can lead to banded leaf blight in maize, rice, and sorghum, cataplasm in cotton, aerial blight, and stem rot in mung beans and soybeans, as well as sheath rot, cabbage heart rot, potato black spot, and leaf blight (Yang and Li, 2012). *R. solani* belongs to soil habitative fungi. Once it has colonized a certain field, it can survive in the soil for many years in the form of sclerotia (Ajayi-Oyetunde and Bradley, 2018). At times, the mycelium is capable of overwintering in contaminated soil or diseased debris, and may also persist through the winter in the seeds of its host (Ogoshi, 1987). Currently, *R. solani* can be categorized into 14 anastomosis groups (AG1 to AG13 and AGBI) based on the ability of the mycelia to fuse with one another (Li et al., 2021). Each anastomosis group can be further divided into different anastomosis subgroups depending on the morphology and host range of the mycelia, as well as the physiological, biochemical activity, heredity, and pathogenicity of *R. solani*. In previous reports, AG1 anastomosis groups are often studied as the typical ones with high pathogenicity (Yamamoto et al., 2019; Zhang et al., 2021). However, recent studies indicate that AG4 anastomosis groups are becoming the dominant pathogenic species in various crops, including corn and millet (Sumner and Bell, 1982; Hao et al., 2023).

Chemical control is the primary way to prevent and manage diseases and insect pests in the world, which guarantees food production (Pandit et al., 2022). However, the overuse of chemical pesticides can severely harm the environment and human health (Pathak et al., 2022). Therefore, developing biopesticides is an effective solution to the issue of pesticide pollution, which can replace some chemical pesticides (Jaiswal et al., 2022). Biogenic protein elicitors typically arise from plant-pathogen interactions as a substance that activates the plant defense response (Wei et al., 1992). The elicitin response receptor protein identified in South American potato recognizes and enhances potato resistance to late blight resistance (Du et al., 2015); Flg22 and Pep1 elicitors can induce the pattern-triggered immunity (PTI) response in plants, and the induction pathways are distinct and interdependent (Du et al., 2015). The GH12 family of glycoside hydrolases has been shown to act as elicitors to cause a burst of reactive oxygen and callose deposition, further increasing the expression of plant defense genes and thus inducing an immune response in plants (Wang et al., 2018).

Thaumatin-like proteins (TLPs) are a member of pathogenesis-related (PR) proteins family 5 (PR-5 protein) and are identified by their highly conserved thaumatin domain (de Jesús-Pires et al., 2020). The TLPs have been shown to exhibit antifungal functions, covering inhibition of fungal enzymes (β-glucanase, xylanase, α-amylase, and trypsin), as well as the ability to lyse fungal cell membranes and spores, and to decrease the viability of germinated spores and induce programmed cell death in fungi (Trudel et al., 1998; Liu et al., 2010). However, the function of TLP gene family in plant pathogenic fungi has not been extensively studied. In the present study, we performed a comprehensive identification of the TLP gene family in AG4-JY, which belongs to the AG4 HG-III group and whose genome has recently been published (Liu Y. et al., 2023), and analyzed its composition and evolutionary relationships in different AGs. We also evaluated its secretory activity and the hydrolysis of glycoside hydrolase, and conducted a preliminary exploration of the possibilities for the application of RsTLP3 as a plant elicitor.

## Materials and methods

### Bioinformatics prediction and analysis of thaumatin-like family proteins

The hmmsearch program of HMMER^1^ and the Hidden Markov profile PF00314 from the InterPro database were used to predict the TLP in *R. solani* AG4-JY proteome (GenBank accession number: GCA_025504695.1). Furthermore, the candidate TLPs were confirmed using the SMART online tool (Letunic et al., 2021).

WoLF PSORT^2^ was employed to predict the subcellular localization of TLP proteins. The organism type was selected as fungi (Horton et al., 2007).

SignalP 5.0^3^ was used to predict the N-terminal signal peptide of TLPs protein sequences (Almagro Armenteros et al., 2019).

### Phylogenetic analyses of the TLP of *R. solani*

To analyze the phylogenetic relationships of the TLP gene family in different anastomosis groups of *R. solani*, we first identified members of the TLP gene family in AG1-1A, AG1-1B, AG1-1C, AG2-IIIB, AG3-1AP, AG4-HG-I, AG5, AG6, and AG8 using the same method described above for the identification of the TLP gene family in AG4-JY. The phylogenetic tree of TLP proteins was constructed using FastTree2 software with the maximum likelihood method (Price et al., 2010).

### Extraction the total RNA of *R. solani*

*R. solani* mycelium was cultured on Potato Dextrose Agar (PDA, Lanbo Biotech, China) medium for seven days, Afterward, it was ground in a pre-cooled mortar, and collected in RNase-free centrifuge tubes. The samples were frozen and mixed with 1 mL of TransZol (TIANGEN Biotech, China) by vortexing. Next, 200 μL of chloroform was added, and the mixture was vortexed again and left on ice for 5 min before being centrifuged at 12,000 rpm for 10 min at 4°C. The supernatant was transferred to a new RNase-free centrifuge tube. To extract the RNA, 300 μL of isopropanol was added to the mixture and the mixture was centrifuged at −20°C for 20 min at 12,000 rpm. After centrifugation, the supernatant was discarded and the precipitate was washed with 1 mL of 75% ethanol, before being centrifuged again. The supernatant was removed and the precipitate was left on ice for 10 min to allow the alcohol to evaporate completely. Next, 30 μL of RNase-free ddH_2_O was added to the precipitate, which was dissolved at a 55°C in water bath. Finally, the RNA was stored at −80°C for future use.

### Yeast secretion system that verified the signal peptide

The signal peptide sequences of the TLP genes were amplified using *R. solani* cDNA as the template. Subsequently, the amplified sequences were used to construct a recombinant plasmid with the pSUC2T7M13ORI vector (preserved by the Key Laboratory of Plant Physiology and Molecular Pathology in Hebei Province), employing *Eco*RI and *Xho*I restriction sites, respectively. Invertase-negative yeast strain YTK12 colonies were selected from yeast extract peptone dextrose medium solid plates (YPD, Solarbio, China) and transferred to 5 mL of YPD liquid medium. The culture was incubated for three days at 30°C with shaking at 220 rpm. On the following day, the culture was diluted to an optical density of approximately OD550 = 0.2 and incubated for an additional 3–4 h until the yeast cells reached the logarithmic growth phase. The transformation system was prepared by mixing 240 μL of 50% PEG3350 (240 μL, Beijing Solarbio, China), 36 μL of lithium acetate (Sigma-Aldrich, USA), ssDNA, 2 μL of plasmid, and 72 μL of ddH_2_O. The mixture was thoroughly mixed and left at 25°C for 30 min. The transformation system was incubated at 42°C for 15 min and subsequently cooled on ice for 5 min. After centrifugation at 3,400 rpm for 4 min, the yeast cells were suspended in 200 μL of ddH_2_O. The suspension was applied to a salmonella Shigella/-Trp with Agar (CMD-W, LABLEAD, China) solid plate and cultured in an incubator (Saifu, China) at 30°C for 3 days. The secondary screened the transformants, respectively, carrying pSUC2-SP, pSUC2-Mg87 (negative control), and pSUC2-Avr1b (positive control) were inoculated into 5 mL of liquid CMD-W medium and cultured at 30°C and 250 rpm for 36 h. Meanwhile, YTK12 was inoculated with 5 mL of YPD liquid medium. The disaccharide utilization capacity of these yeast transformants was determined by diluting the culture to the same concentration and incubating them at 30°C with the same amount on CMD-W, YPR agar with antimycin A (YPRAA, Solarbio, China), and sucrose medium, respectively (Yang et al., 2020).

The transformants of YTK12 was re-suspended in 750 μL of ddH_2_O. Subsequently, 250 μL of 10 mM sodium acetate buffer (pH = 4.7) and 500 μL of 10% sucrose solution were added. The mixture was incubated at 37°C for 10 min. After centrifugation, approximately 100 μL of supernatant was transferred to a cuvette infused with 900 μL of 0.1% 2,3,5-triphenyltetrazolium chloride (TTC, Aladdin, China) solution. The solution was allowed to react at 25°C for 5 min, after which the resulting change in color was observed to determine the invertase enzymatic activity.

### Prokaryotic expression of TLP family proteins

The pET28a-SUMO (preserved by the Key Laboratory of Plant Physiology and Molecular Pathology in Hebei Province) cloning vector was constructed as follows: the *R. solani* cDNA was used as the template and the signal peptide and stop codons of each *TLPs* were removed. *Not*I and *Bam*HI were selected as the restriction sites to construct the pET28a-SUMO recombinant expression vector. The DH5α (TransGen Biotech, China) was transformed in accordance with the instructions and positive clones were screened and verified. Single colonies of positive clones were selected in 500 μL of Luria-Bertani (LB, Solarbio, China) medium containing chloramphenicol and Kanamycin (Sangon Biotech, China). The solution was shaken for 3–5 h and then 100 μL of it was transferred to 10 mL of fresh LB medium. The mixture was shaken overnight at 37°C and 220 rpm. The OD600 of the culture was adjusted to 0.2 by transferring it into 100 mL of resistant medium. The bacterial suspension was shaken until the OD600 was less than 0.6. After that, arabinose was added to the culture at a final concentration of 2 mM to induce it for 1 h at 37°C. Finally, isopropyl β-D-1-thiogalactopyranoside (IPTG, Coolaber, China) was added to the culture at a final concentration of 1 mM to induce it overnight at 28°C. Bacteria expressing TLP genes were collected before and after the induction with IPTG as the inducer. The collected bacteria were added to 1 × buffer and boiled at 95°C for 5 min. After centrifugation for 1 min, 10 μL of supernatant was taken, and the protein induction was detected through SDS-PAGE.

### Enzymatic activity detection of TLP family

The enzyme activity of TLPs was measured using the Plant β-glucosidase ELISA kit (Tianjing RUICHUANG Bio-Technology). Standard wells and test wells were set up according to the following method: For the standard wells, 50 μL of standards with varying concentrations were added to each well. For the test wells, 10 μL of the sample to be tested was added first, followed by 40 μL of sample diluent. Then, 100 μL of horseradish peroxidase-labeled detection antibody was added to both the standard and test wells. The reaction wells were sealed with a sealing membrane and incubated at 37°C for 60 min, followed by discarding the liquid. Subsequently, 50 μL of substrate A and B were added to each well, and the mixture was incubated at 37°C for 15 min in darkness at room temperature. Finally, the termination solution was added, and the OD value was measured at 450 nm within 15 min. Data were obtained from triple independent replicates, and statistical significance was determined using *t*-test.

### Analysis of disease resistance induced by TLP proteins

The supernatants of *E. coli* transformants expressing TLP proteins and the control strain carrying the original vector were sonicated, respectively. The collected cells of each strain were re-suspended in the lysis buffer. The ultrasonic cell disruptor was set to 75 W and operated using ultrasound for 5 s, followed by a 3-s pause, with a total treatment time of 30 min. All cell lysates were centrifuged at 12,000 rpm for 10 min to extract soluble proteins. The crude protein solution was dropped on maize leaves (B73, 4–6 leaf stage), which were maintained in a growth chamber with a temperature range of 25–30°C and a relative humidity (RH) between 50 and 100% throughout the day and night. Once the solution on the leaves had dried, *R. solani* AG4-JY disks were inoculated onto the leaves. The inoculated plants were incubated at 25°C with 100% RH for 24 h. They were maintained under the same conditions as before infection, and the development of leaf lesions were observed after one week. The lesions on the maize leaves were measured using ImageJ software^4^ (Schindelin et al., 2015). The mean and standard error were calculated using Graphpad software, and a *t*-test was performed to analyze the significance of the data. Samples were collected and rapidly frozen in liquid nitrogen, and stored at −80°C for subsequent experiments.

### Effects of TLP family proteins on defense gene expression in maize

The primers used for quantitative analysis of maize defense genes were designed using NCBI (Supplementary Table 1). RNA was extracted from the inoculated leaves mentioned above. The reverse transcription system comprised 4.0 μL of UEIris II RT-PCR MasterMix (Biorigin, China), 2.0 μL of RNA, 1.0 μL of dsDNase, and 13.0 μL of RNase-free water. The polymerase chain reaction (PCR) procedure employed the Bio-Rad thermal cycler (USA) and was conducted as follows: 37°C for 2 s, 55°C for 10 m, and 85°C for 10 s. The synthesized cDNA was quantified and diluted to a concentration of 200 μg/μL. The gene expression level was validated using 2 × AugeGreen™ Master Mix (UElandy, China) and Bio-Rad cfx96 (Bio-Rad, USA) for the gene expression level. The RT-qPCR program settings were as follows: the first step was performed at 95°C for 2 min, followed by 45 cycles of denaturation at 95°C for 15 s and extension at 60°C for 1 min each. The *Actin* gene was used as the reference gene, and the 2^–△△Ct^ method was used to calculate the relative expression level of target genes. Data were obtained from triple independent replicates, and statistical significance was determined using *t*-test.

## Results

### Acquisition of the members of TLP family in *R. solani* AG4-JY

**FIGURE 1:**
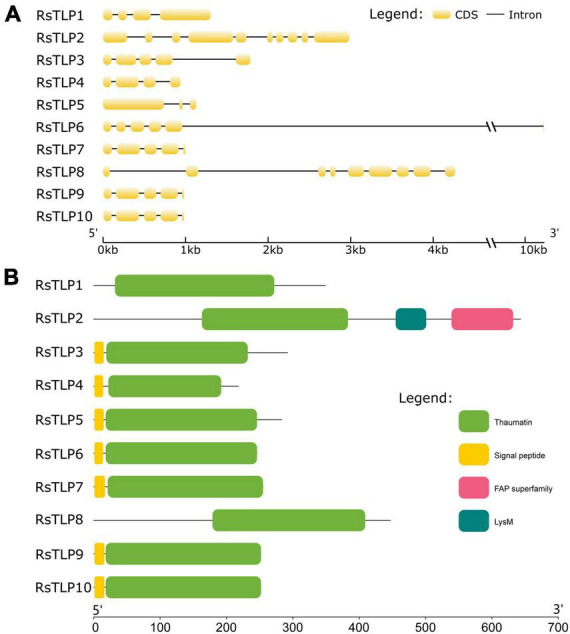
Analysis of the gene structure and conserved domains of the *RsTLPs*. **(A)** The gene structure of *RsTLPs*. **(B)** The protein domain of RsTLPs.

To investigate the role of TLP proteins in fungi, we conducted a systematic identification of the TLP gene family in *R. solani* AG4-JY using hmmsearch. We identified ten genes that contained the thaumatin domain. Further, the candidate proteins were subjected to the SMART online tool to confirm the thaumatin domain. The result showed that all the 10 members containing the thaumatin domain, and belonged to the thaumatin family (RsTLP) (Table 1). Furthermore, the structural of *RsTLP* genes was analyzed and the results revealed that they had between 3 (*RsTLP5*) and 38 (*RsTLP10*) exons (Figure 1A). In particular, there are four tandem repeats of the TLP gene (RsTLP3, RsTLP4, TLP5, and RsTLP6) located on chromosome 7. The CDS sequences of *RsTLP9* and *RsTLP10* exhibit a six-base difference, yet they share an identical protein sequence (Supplementary Table 2). In addition, seven proteins were identified as having signal peptides and lacking a transmembrane domain, indicating their potential as secreted proteins (Figure 1B and Supplementary Table 2).

**TABLE 1:** The member of thaumatin-like protein family in AG4-JY.

Gene	Name	Position	Strand	Protein location	Signal peptide
EVM0004068	RsTLP1	Chr04:1107621-1108924	–	Extracellular	NA
EVM0004263	RsTLP2	Chr06:2338832-2341816	+	Extracellular	NA
EVM0009456	RsTLP3	Chr07:2647508-2649295	+	Extracellular	1∼16
EVM0009537	RsTLP4	Chr07:2649394-2650333	+	Extracellular	1∼15
EVM0001778	RsTLP5	Chr07:2651313-2652443	+	Extracellular	1∼15
EVM0001349	RsTLP6	Chr07:2653053-2663285	+	Extracellular	1∼15
EVM0004822	RsTLP7	Chr11:1143980-1144975	–	Extracellular	1∼18
EVM0003710	RsTLP8	Chr11:1196462-1200728	+	Extracellular	NA
EVM0003570	RsTLP9	Chr12:144600-145574	+	Extracellular	1∼15
EVM0009733	RsTLP10	Chr12:220074-221048	–	Extracellular	1∼15

### Phylogenetic analyses of different AGs TLP gene family in *R. solani*

**FIGURE 2:**
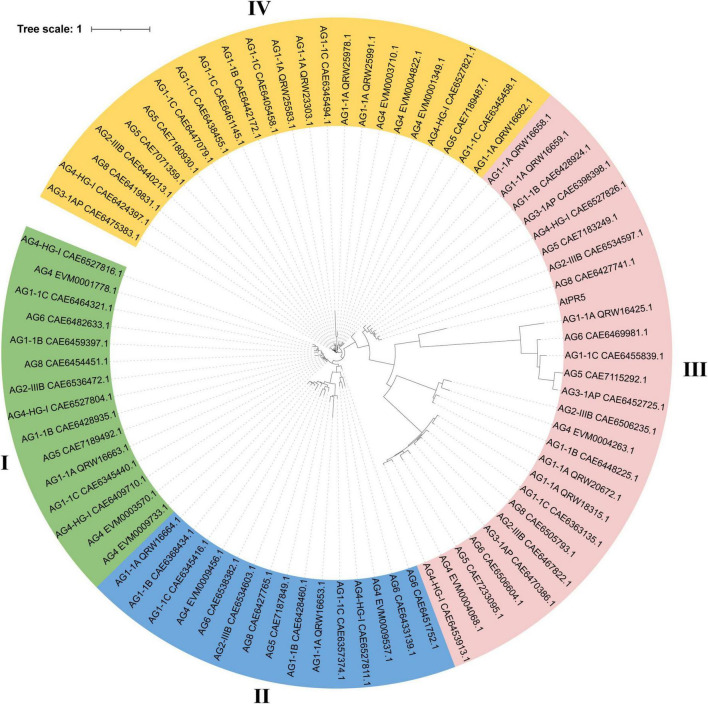
Phylogenetic relationships of thaumatin-like proteins (TLPs) in 10 anastomosis groups of *Rhizoctonia solani*. The tree was constructed using FastTree2 software with the maximum likelihood method. Each protein ID in the tree consists of the anastomosis group name and the GeneBank ID of the TLP protein. Different color represent different clade of TLP proteins.

To analyze the evolutionary relationship of the TLP gene family in different AGs of *R. solani* and understand its relationship with the TLP gene family in AG4-JY, we identified TLP gene families in AG1-1A, AG1-1B, AG1-1C, AG2-IIIB, AG3-1AP, AG4-HG-I, AG5, AG6 and AG8 using the same method as above. The TLP genes were present in all AGs, with the number ranging from 4 to 12. AG3 had 4 genes and AG1-1C had 12 genes (Supplementary Table 3). In addition, the number of these varies between different strains of the same AG. For example, within AG1, the range is 7 to 13, and within AG4, the range is 8 to 10. Furthermore, the protein sequences of the TLPs from different AGs and AtPR5, the TLPs protein of Arabidopsis, were analyzed using the maximum likelihood method (bootstrap = 1,000). We found that the TLP genes can be categorized into four different classes, with the TLP genes in AG3 distributed only in classes III and IV, including three in III and one in IV (Figure 2). The TLP genes in other AGs were allocated to the four classes, indicating their evolutionarily conserved. Overall, the evolutionary relationship of TLP genes was consistent with the AGs. For instance, strains belonging to the same anastomosis group tended to cluster together, and anastomosis groups AG2-IIIB, AG3-1AP, AG5, and AG8 clustered together. Furthermore, we found that the third class had a long evolutionary distance which included the TLP gene AtPR5 in Arabidopsis, This suggests that the genes in this class are relatively old and predate the differentiation of fungi and plants.

### Verification of the secretion activity of the TLP family proteins

**FIGURE 3:**
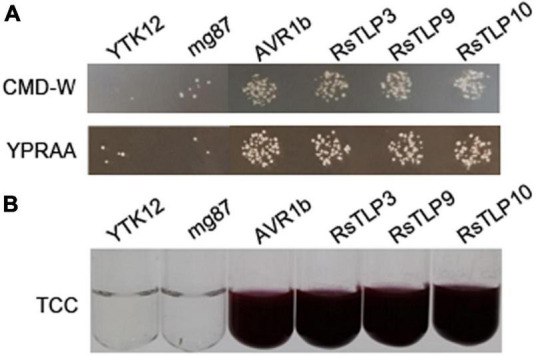
Functional evaluation of the signal peptide of the 3 candidate TLPs. **(A)** the secretion function of the signal peptide of RsTLP3, RsTLP9 and RsTLP10 were confirmed using the *Saccharomyces cerevisiae* strain YTK12 containing the pSuc2t7M13ori vector, Avr1b was used as the positive control and mg87 as the negative control. The yeast transformants grow exclusively on CMD-W and YPRAA medium, demonstrating the presence of active invertase secretion in the system. **(B)** invertase activity was detected by reducing 2,3,5-triphenyltetrazolium chloride (TTC) to the insoluble, red colored 1,3,5-triphenylformazan (TPF).

The yeast secretion experiment was used to verify the secretory activity of the above-mentioned candidate secretion proteins of the TLP family. Among them, Avr1b was used as a positive control and mg87 as a negative control. The results showed that three (TLP3, TLP9 and TLP10) out of the 10 candidate secreted proteins had secretory activity (Figure 3). These finding suggest that these secreted proteins may have a significant impact on the interactions between the AG4-JY and its host.

### Assay for the enzymatic activity of TLP family proteins

**FIGURE 4:**
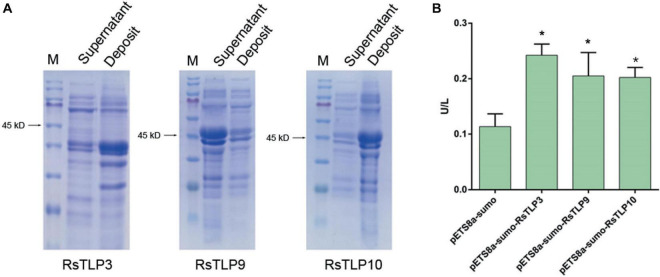
Detection of glycoside hydrolase enzyme activity in TLPs. **(A)** Expression of RsTLP3, RsTLP9 and RsTLP10 proteins after ultrasonic fragmentation. Prokaryotic expression of the target proteins by fusing with sumo-tag and the protein size is about 45 kD. The bands of gel electrophoresis was stained with trypan blue. The M represent the Blue Plus^®^ IV protein marker (10–180 kDa, TransGen Biotech). **(B)** Analysis of RsTLP3, RsTLP9 and RsTLP10 glycoside hydrolase activity using enzyme-linked immunosorbent assay. Error bars represent standard error of the mean (*n* = 3). *P < 0.05.

To determine if the secreted proteins RsTLP3, RsTLP9, and RsTLP10 possess glycoside hydrolase activity, we expressed them using a prokaryotic expression system. Plasmids were constructed for each gene and transferred to BL21 cells, which were induced for expression at 37°C, 28°C, and 25°C. SDS-PAGE protein gels showed that the target bands (approximately 45 kD) were induced at all three temperatures. However, the proteins obtained were primarily present in the form of inclusion bodies within the lysate precipitates of the expression host bacterium. The expression of these proteins was induced further using host bacteria containing molecular chaperones. Successful protein expression was demonstrated by SDS-PAGE. Ultrasonic fragmentation revealed that protein RsTLP9 was solubilised in the supernatant but expressed at lower levels than in the precipitate. Protein RsTLP3 was widely distributed in the supernatant, while protein RsTLP10 was expressed at very low levels in the supernatant (Figure 4A). The proteins were subsequently purified to assess their enzymatic activity. The enzyme activity of RsTLP3 was 0.2424 U/L, RsTLP9 was 0.205116 U/L, and RsTLP10 was 0.202248 U/L (Figure 4B), indicating that these proteins have glycoside hydrolase activity. This result is further evidence that these three proteins are members of the TLP family.

### Analyzing TLP family proteins involved in inducing disease resistance in maize

**FIGURE 5:**
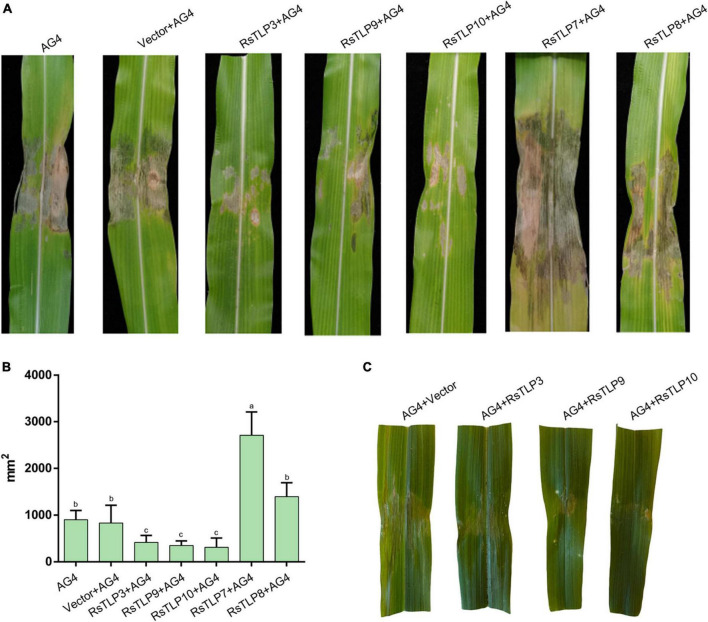
Analysis of disease resistance induced by TLPs. **(A)** After inducing the expression of the target protein, the lysate of each strain was sprayed onto maize leaves and inoculated with *R. solani*. **(B)** The mean size of the sheath blight lesion. **(C)** After three days of inoculation with the *R. solani*, the lysates of each strain were sprayed onto maize leaves. AG4: AG4-JY strain only; AG4-Vector: AG4-JY strain with lysate containing an empty vector; AG4-RsTLP3: AG4-JY strain with lysate containing the RsTLP3 protein; AG4-RsTLP3: AG4-JY strain with lysate containing the RsTLP3 protein; AG4-RsTLP9: AG4-JY strain with lysate containing the RsTLP9 protein; AG4-RsTLP10: AG4-JY strain with lysate containing the RsTLP10 protein; AG4-RsTLP7: AG4-JY strain with lysate containing the RsTLP7 protein; AG4-RsTLP8: AG4-JY strain with lysate containing the RsTLP8 protein. Different letters above the bars indicate values are significantly different from each other (*t*-test, *p* < 0.05).

To assess the potential of these proteins as elicitors of plant immunity, we sprayed the lysates of each strain onto maize leaves after inducing expression of the target proteins. The leaves were inoculated with AG4-JY to evaluate their response. The effects of the target proteins on the maize leaves were analyzed after one week. The results showed that the three proteins, RsTLP3, RsTLP9 and RsTLP10, were able to significantly reduce the blight symptoms compared to the control and the lysates with the empty vector (Figure 5). To further verify the role of RsTLP3, RsTLP9 and RsTLP10 in plant response to disease, we sprayed those on maize leaf two days after inoculation with AG4-JY and observed the spots 3 days later. The results showed a significant reduction in the size of the spots on the sprayed leaves compared to the unsprayed leaves, suggesting that RsTLP3, RsTLP9 and RsTLP10, play a role in plant response to pathogen infection. Furthermore, we also investigated whether the non-secretory proteins (RsTLP7 and RsTLP8) also played a role in reducing the pathogenicity of the pathogen. The results showed that they were not able to reduce the size of the lesions. These results indicate that GH64 family proteins with secretory activity have the activity to induce sheath wilt resistance in maize.

### Effects of TLP family glycoside hydrolases on defense gene expression in maize

**FIGURE 6:**
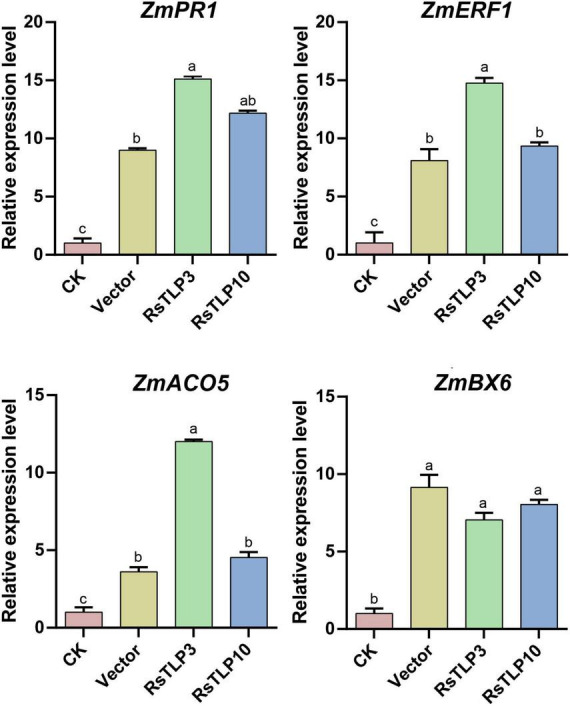
qRT-PCR analysis of the relative expression of related genes in inoculated with *R. solani* after spraying with different proteins. CK, control; vector, lysate containing an empty vector; RsTLP3, lysate containing the RsTLP3 protein; RsTLP10, lysate containing the RsTLP10 protein. Different letters above the bars indicate values are significantly different from each other (*t*-test, *p* < 0.05).

Previous research suggests that plant immune attractants can activate immune signaling pathways such as jasmonic acid (JA), salicylic acid (SA) and ethylene to enhance plant resistance to disease and insect pests. As RsTLP3 and RsTLP10 were found to be the most effective in inducing disease resistance in maize, we analyzed genes associated with the immune response in maize by spraying the lysates of strains containing RsTLP3 and RsTLP10 protein, respectively. These genes included the *ZmPR1* gene of the SA pathway, the *ZmBX6* gene of the JA pathway, and *ZmACO* and *ZmERF1* of the ethylene pathway. The results showed that the expression of *ZmPR1* gene showed significant up-regulation in all treatments, with 8.99-fold up-regulation by spraying the lysates with the empty vector, 15.1-fold up-regulation by spraying the lysates with RsTLP3, and 12.18-fold up-regulation by spraying the lysates with RsTLP10 (Figure 6). In addition, the expression of *ZmACO* and *ZmERF1* genes were also up-regulated, with increases of 3.61, 12.01 and 4.53 times compared to the control, respectively (Figure 6). However, there was no significant difference in BX6 expression between the sprayed vector and RsTLP3 and RsTLP10 (Figure 6). These results suggest that RsTLP3 primarily enhances disease resistance in maize by inducing the expression of genes related to the SA pathway and ethylene.

## Discussion

TLPs are a crucial protein family in plants, playing a significant role in host defense and developmental processes (Liu et al., 2010). They belong to the Pathogenesis related 5 protein family in plant, and angiosperms exhibit a high degree of diversity in these proteins (Zribi et al., 2021). TLPs are primarily associated with responses to biotic stresses, although some predictions suggest their involvement in low temperature, drought and osmotic stresses (Petre et al., 2011; Muoki et al., 2012; Park and Kim, 2021; Liu Q. et al., 2023). Numerous studies have indicated that TLPs exhibit antifungal activity by permeabilising the fungal cell membrane (Vigers et al., 1992). This activity is believed to be associated with their capacity to bind and degrade glucan molecules, as well as inhibit xylanase enzymes (Fierens et al., 2007). TLPs are present not only in plants but also in fungi (Grenier et al., 2000; Osherov et al., 2002; Greenstein et al., 2006; Sakamoto et al., 2006). TLP gene families have been identified in several basidiomycete fungi, including *Irpex lacteus*, *Lentinula edodes* and *R. solani*, as well as in the ascomycete fungus *Aspergillus nidulans*. Fungal TLP gene families must meet two criteria: they must have homology with plant TLP family genes, and they must have the function of hydrolyzing β-1,3-glucanases (Grenier et al., 2000). However, a systematic identification of TLP gene families in the whole fungal genome has not yet been conducted. In the present study, the TLP gene family in *R. solani* AG4-JY was identified and analyzed. A total of 10 TLP family genes were found in AG4-JY. Additionally, proteins for five of these genes were obtained using a prokaryotic expression system. It was discovered that all five proteins had endo-1,3-glucanase activity, which confirms their classification as TLP proteins. These findings suggest that the identified genes in AG4-JY are members of the TLP gene family. Furthermore, we discovered that the TLP genes were present in all AGs of *R. solani*, with their numbers ranging from 4 to 12. Specifically, AG3 had 4 genes while AG1-1C had 12 genes. These findings highlight the significant roles played by TLP genes in *R. solani*.

Secreted proteins play a crucial role in the interactions between plant pathogenic fungi and their hosts (Lo Presti et al., 2015; Jia et al., 2023). Numerous secreted proteins have been identified in plant pathogenic fungi. Their expression is tightly regulated during pathogen invasion, resulting in successful infection (Toruño et al., 2016). The genome of AG4-JY contains approximately 709 functionally diverse secreted proteins that may contribute to the process of *R. solani* infection (Liu Y. et al., 2023). The results of our experiments indicate that three of the ten TLP proteins, namely RsTLP3, RsTLP9 and RsTLP10, exhibit secretory activity and that all three genes possess endo-β-1,3-glucanase activity, suggesting their crucial role in the pathogen’s infection process. However, further research is required to comprehend the precise function of these genes.

Traditional fungicides focus primarily on pathogen-specific genes or pathways to control disease in crops. The triazoles are sterol biosynthesis inhibitors target (Leadbeater, 2015). N′-(3-bromo-4-hydroxybenzylidene)-2-methylbenzohydrazide and 3-bromo-N′-(3-bromo-4-hydroxy- benzylidene) benzohydrazide can inhibits the activity of fungal glucosylceramide synthesis (Mor et al., 2015). Mandipropamid targets the cellulose synthase-like PiCesA3. It inhibits cell wall biosynthesis in the *Phytophthora infestans* (Blum et al., 2010). Quinoxyfen (5,7-dichloro-4-quinolyl 4-fluorophenyl ether) effectively controls powdery mildew pathogens, including *Blumeria graminis*, by disrupting the activity of serine esterase (Lee et al., 2008). Dicarboximide fungicides exhibit potent antifungal properties against various ascomycetes, such as *Botrytis* spp and *Bipolaris* spp by targeting the MAP kinase in the high osmolarity glycerol (HOG) pathway (Chihiro and Kosuke, 2010). While, the primary function of plant immune-eliciting is to trigger activation of the plant’s immune system, such as the SA and JA pathways, thereby enhancing disease resistance (Nürnberger, 1999; Thakur and Sohal, 2013; Dalio et al., 2017; Abdul Malik et al., 2020). This application has demonstrated significant benefit in agricultural production. For instance, elicitors such as flg22 and Pep1 can induce PTI response in plants, with distinct and interdependent induction pathways (Bigeard et al., 2015). Furthermore, proteins found in fungi can serve as immune-eliciting agents for plants. For instance, the XEG1 enzyme, belonging to the GH12 family of *P. sojae*, has the ability to interact with receptors on the cell membrane of plants, thereby triggering the activation of plant immunity (Ma et al., 2015). For the TLP genes, they can be triggered by signaling compounds like SA, JA, and ethylene, and enhance the antimicrobial activity (Van Damme et al., 2002). Our study found that TLP genes in AG4-JY can effectively enhance maize resistance to *R. solani* and can induce the *ZmPR1* gene of the SA pathway, the *ZmACO* gene of the ethylene pathway and its downstream *ZmERF1* gene, but have less effect on the related genes of the JA pathway, which is thought to play its role in inducing disease resistance mainly through the SA and ethylene resistance pathways, and its mechanism still needs to be further studied.

## Conclusion

In summary, this study utilized bioinformatics methods to identify and analyze the composition of the TLP protein family members in strain AG4-JY of *R. solani*. The evolutionary relationships and constitution of the TLP family in the different AGs of *R. solani* were also analyzed. Furthermore, the secretion activity and endo-β-1,3-glucanase activity of the TLP family members were verified. RsTLP3, RsTLP9 and RsTLP10 were found to possess these features. Importantly, our study has shown that RsTLP3 can induce disease resistance in maize, indicating its potential as a biopesticide. This research provides a foundation for the study of TLP genes in fungi and serves as a resource for the development of biopesticides.

## Data availability statement

The original contributions presented in this study are included in this article/Supplementary material, further inquiries can be directed to the corresponding authors.

## Author contributions

JL: Formal analysis, Investigation, Software, Visualization, Writing – original draft. SF: Methodology, Writing – original draft. TL: Formal analysis, Software, Writing – original draft. YM: Methodology, Writing – original draft. SS: Formal analysis, Visualization, Writing – original draft. YL: Conceptualization, Formal analysis, Investigation, Project administration, Writing – original draft, Writing – review & editing. ZH: Conceptualization, Formal analysis, Investigation, Project administration, Supervision, Writing – original draft, Writing – review & editing. ZL: Conceptualization, Funding acquisition, Project administration, Supervision, Writing – review & editing.

## Footnotes

1
http://hmmer.org/

2
https://wolfpsort.hgc.jp

3
https://services.healthtech.dtu.dk/services/SignalP-5.0/

4
https://imagej.net/software/imagej/

## Supplementary material

Supplementary Table 1 The primers used in this study.

Supplementary Table 2 The protein sequences of the RsTLPs.

Supplementary Table 3 The member of thaumatin-like proteins in different anastomosis groups.
