# Supplementary material for: Molecular characterization revealed the role of thaumatin-like proteins of Rhizoctonia solani AG4-JY in inducing maize disease resistance
Source: Front Microbiol. 2024 May 15;15:1377726. doi: 10.3389/fmicb.2024.1377726 (PMC11135045; doi:10.3389/fmicb.2024.1377726)
Supplement: Supplementary Table 2 — The protein sequences of the RsTLPs. [file Table_2.DOCX]

> RsTLP1 CDS

ATGACATCGAACTCTGGACGCATTCTGGTACTCAGAGGACTTATCCTTGCGGCACATATCGCTGTAACGAGTGCTCGACGATGGACTGTCAACAACGACTGCCCTTTTACCATTTGGCCGGCCATATACACCAGTAATGCCTCTGCGGTCACTCTAAGCGGTGTTGAAACCGGCTGGGAAGCGGCTCCCAACAGCTCGCGCTCTTTTGTTGTCCCAGAGGGTTGGACTGGAGGATACATATGGGGACGCCGAGATTGCAACTTTTCCGTGGGGAATAACCCAGCTGATACAAATACGACCACTGGAGGATGTGTTTCAGGGGGATGCCCGGGTGGATTGTATTGCACAGCCCTGGGCTCATCGCCCACGACACATGCCGAATGGACTCTAGCTCCCGGAGATGGCTCCGGTGCTGATTACTATGATATCTCAATAGTCCAAGGTTTTAACTTGCCTATGCAAGTCGTACCAAGCGCAATGGGATGTGGAATTGCTGAATGCGCAGTGGATCTTAATGCGAATTGCCCAGACCCCCTCCGTGGACCTTTTGCTCCTAACACTAACAACACAGTACCAATCGGTTGCAAGTCAGCATGCACCGCAAACCTTGATGGAAATCCAGATAATAGTGCTGCGTGCTGTACGGGCGAATTCGCAGCGCAAGGTGCATGCCCAACCACTGGCGTTCCATTTTACGATTACTTCAAGAAGGCTTGTCCTTTTACATACGCTTATTCACAGGATGTGACTTCGGGTACGGCTTTACAGACTTGCCCCGGCTCTAGCTACCCGGATTATACTGTCACCTTTTGTCCTCCTGCGGTGCTCGCATCTAACTCAACAACTCAGAGTAGCGGCCCCGTGGCTACATCGGGAACAATTACTGGCATCCCTTCCATGACCAATGTACCAACGAGTGCACTCACTTCATCAAGAAGTTTAGGGACATCTTTGATTCCCTCGAATGCCAACGGCGCCGATACTCTCACTGTGATGCATGTAGGATTGGTCTTGGGGCTTCTGGCCAGCATTTGTGGGTCTATGCCGTAG

>RsTLP1 pep

MTSNSGRILVLRGLILAAHIAVTSARRWTVNNDCPFTIWPAIYTSNASAVTLSGVETGWEAAPNSSRSFVVPEGWTGGYIWGRRDCNFSVGNNPADTNTTTGGCVSGGCPGGLYCTALGSSPTTHAEWTLAPGDGSGADYYDISIVQGFNLPMQVVPSAMGCGIAECAVDLNANCPDPLRGPFAPNTNNTVPIGCKSACTANLDGNPDNSAACCTGEFAAQGACPTTGVPFYDYFKKACPFTYAYSQDVTSGTALQTCPGSSYPDYTVTFCPPAVLASNSTTQSSGPVATSGTITGIPSMTNVPTSALTSSRSLGTSLIPSNANGADTLTVMHVGLVLGLLASICGSMP

> RsTLP2 CDS

ATGAACGAGAAGTTCCCTGGGTATTTCACCGAGCACCCAAATTTGCCTGAGGAAGACTTGAAGAGGTACAAGTCTCAGGAGGCGATTGTGAAACAACTTGTAGAGATCTATCAGAAACCGGATTATTCAGAGGACAATGCGGATATGAACAAGGAAGTGCTTAGACTCATGAACGAGATGCAAGAGCTTGGGTCGCCCCCGACCGAGATTATGGGAGAGGTTCCTCCTGGTTTCGACTTTAATTCTCCAGAAGGCATGGCGAAGATGTTGGATAAAGATGGATGCGTTATTAATCAATCTCAGTCCCCCTCGACTTTTCCTCCATCCGACTTTGACCGATCGTACCTCGATCCGACGGACAGTGCTCATCTACTCAACAACCTTACTATATACGTCTACTCCCACGGCATGAATCTCGTTATACTCAAGCCACGAGGATACTTGGCGTGTGTTTATATGCGGTGGGGCTTTAGCTACGCCCGACTTTTTACAGTTACGAATGCATGCCCGTTCACAATTTGGCCGGGCGTATGGACCAATACCTCTTATGGGACCGCAAAACCATCGGTCGAAGGTGGGTGGGAAGCGCCTGCGAACACCTCGAAGCAATTTGCTGTACCTGATAATTGGGCGGCTGGGCGGATTTGGGCTCGTCGAGGATGTAACTTTGCCAATTCGCAGGGACCAGAGAGTTGTGTTACTGGAGGCTGCGTTGGTGGATTGAATTGTACGGATGCTGGAGTGGCTCCAACGACTTTGGCGGAATGGACACTCTCGCCTACGAATGACGCTGCTGACTACTATGACGTGTCATTGCTGGATGGATTCGATCTCCCTGTGCGTATTACGCCGAGCGCCGAGTGTCCTGTAGCCGAATGTGCAGTTGACCTTGTTGCAAATTGCCCCCAACCTCTGAGGATTCCAACCGACCAGAATCAACCGGCCCAAGGATGCACTACTTCGTGTTTTGCGAATCTGGACGGGAATCCAGGGGATTCTGCCAACTGTTGCACTGGGCACAACATTGCGTTCTTCGATTACTTCCGTGGGTCGTGCCCGGGTGCGTACGCATGGACTTTCGACGGCGTGAACCCTGGGGACGTGCTCAAAACCTGCTCAGGGGCTAACCGAGCTGATTATACTGTTACCGAACCAGCTCGTTTCCGGATGGCCAAGCGACGGCACATTTCACAAAAGGCAAATAGAAGCTGTCATAATGAATATCGGCCCGCCTTGGCAATTCCTTCCCTTTATAGTCCGCCCCCTCCTCCATATTTTTCCAACAATCTCCCTCATTCTCGTCTTATTCTCCTTGTTATTTCCGCGTTGGCTTCGGCTACCTTTGCGTCCGAGTGTACACGTACCTATACCGTCAAGGAAGGTGATTGGTGTGATACTATTAGCGCCGCTAATAATGCCTCTACCTACCAACTCTCCACCATAAACGCAGACAAAATCAATGACGCTTGTACCAACCTCGAAATTGGCCAGCAACTTTGCCTTGGGGCCAACATCAACGCCACAATGCTCTATGCCAACAACCCCCAAATCGACGAGTACTGCAGCAATATCTACATTGGCGAGGTCCTTTGTGTAGCTGGTGCCTATGCGGCCCCAGAGCCTATTCCTGAACGTCAAGTCGGTGCCCCTGGAGGCGAACCTGCTGGTCCTCCTGCTACCACCCCTTACCCAGAAGCCAAGCCCAAGACCCAGCCCAAGGTCAATGACCCGGCTCCCGCCCCGGAAAACAACACTCCGGCCTCGAGCAACAACACTCCGGCTTCGACTAACAACATTCCCACTCCGGCCAACAACGAGCCGGCTGCCACTCCTGCGCCCGAGAATAACCAGTCCGCCACCCCTGCGCCCGACAGTGAGAATGACGACAACCTTCCCGAGTGCGACGACCCCAACGATGATGGTTACTACTAA

>RsTLP2

MNEKFPGYFTEHPNLPEEDLKRYKSQEAIVKQLVEIYQKPDYSEDNADMNKEVLRLMNEMQELGSPPTEIMGEVPPGFDFNSPEGMAKMLDKDGCVINQSQSPSTFPPSDFDRSYLDPTDSAHLLNNLTIYVYSHGMNLVILKPRGYLACVYMRWGFSYARLFTVTNACPFTIWPGVWTNTSYGTAKPSVEGGWEAPANTSKQFAVPDNWAAGRIWARRGCNFANSQGPESCVTGGCVGGLNCTDAGVAPTTLAEWTLSPTNDAADYYDVSLLDGFDLPVRITPSAECPVAECAVDLVANCPQPLRIPTDQNQPAQGCTTSCFANLDGNPGDSANCCTGHNIAFFDYFRGSCPGAYAWTFDGVNPGDVLKTCSGANRADYTVTEPARFRMAKRRHISQKANRSCHNEYRPALAIPSLYSPPPPPYFSNNLPHSRLILLVISALASATFASECTRTYTVKEGDWCDTISAANNASTYQLSTINADKINDACTNLEIGQQLCLGANINATMLYANNPQIDEYCSNIYIGEVLCVAGAYAAPEPIPERQVGAPGGEPAGPPATTPYPEAKPKTQPKVNDPAPAPENNTPASSNNTPASTNNIPTPANNEPAATPAPENNQSATPAPDSENDDNLPECDDPNDDGYY

> RsTLP3 CDS

ATGAAGACTGCTGTTATCCTTGCTGCTATCGCCGGCTCTGCTCTCGGTCGTACATTCACGGTCTATAATGCATGCCCATTCACTGTTTGGCCCGCTATCTTCACTGACCTGAACGTTGGCTCGGCTGTTCCCGCAATCGAAAACGGATGGGAAGCACCAGCGTTCTCAAAGCGCTCCTTCACCGTTCCTGACAACTGGAAAGCCGGACGCATCTGGGGTCGCACCCAATGTGATTTCTCCCAGAACCCAGGAATCAAGTCCTGTCTCACTGGTGGATGTAATGGTGGATTGGTGTGCGATAACAAGACTGGGGTTGGCCTTCCGCCGGTCACTTTTGCCGAGTTTACCTTGGTCGACGGGTTCAACTTGCCGATGCGTATCTCGAACAATGGTGGATGCGGAACTGCCGAGTGCACCGTGAACCTCAACAAGGATTGCCCTGAGCCTCTTAGGGGACCTTTGGACACCAATGGGGATGTCGCTGCCTGCAAGTCCGCCTGCTTGGCTCAGATCGATAACCCGTCCGATTCTCCAAGCTGCTGCACCGGCGGGTTCCAGAAACCTTCAACCTGCCCTGCCTCCGGCGTCTTGTATTATGCCTTCTTCAAGAACCGATGCCCGAATTCCTATGTATATGCTTACGACGAATCAAGCGGTACTGCATTGTGGACTTGCCCTTCGTCGAAGAAGGTCGATTGTAACGAGTCAAGCCACATTAAAAATCTCGTCATAGAAATGTTTACTTGTCTGTACATTCGTAACTTGGATGTTATCAATCTACTCGGCTCTCCCCGAGACTTTCTTGAATCGCTTCATACTTGGCGAGGATTCCCACCCATCTATTGCAGCGACGTCCCCGGGTATGCTACAAACTCATGA

>RsTLP3 pep

MKTAVILAAIAGSALGRTFTVYNACPFTVWPAIFTDLNVGSAVPAIENGWEAPAFSKRSFTVPDNWKAGRIWGRTQCDFSQNPGIKSCLTGGCNGGLVCDNKTGVGLPPVTFAEFTLVDGFNLPMRISNNGGCGTAECTVNLNKDCPEPLRGPLDTNGDVAACKSACLAQIDNPSDSPSCCTGGFQKPSTCPASGVLYYAFFKNRCPNSYVYAYDESSGTALWTCPSSKKVDCNESSHIKNLVIEMFTCLYIRNLDVINLLGSPRDFLESLHTWRGFPPIYCSDVPGYATNS

> RsTLP4 CDS

ATGAAATCTGCCGTGCTCCTTACTTGCGCTGGCGTGGTTCTCGGCCGTACCTTCACCGTCTACAATGCGTGCCCATATACTATTTGGCCCGCTATCTTTACTGACCTCAATGTCGGCGCCTCCGTTCCCGCGATCGAAACTGGCTGGGAAGCCCCTGCTAATTCGAAACGAACATTTTCCGTTCCCGAAAACTGGGCGGCCGGTCGTATTTGGGGTCGTACGGAGTGTGATTTTTCAAGCAAGTCTGGCCAGGCTGCTTGCCTGACGGGTGGATGCGACGGTGGCTTAGAGTGCGATACCAAGACTGGGTCTGGTGTCGGGCCGGTTAGCGTTGCCGAATGGACGCTGGGCGCAAGTGATGGTCATGATTGGTACGATGTGAGCCTGGTTGATGGGTTCAACATACCCATGCGCATCTCCAACAACGTCGGTTGCGCGATTGCTGAGTGTGCGGTTAATCTCAATCAAGGCTGTCCTGATGTACTCCAGGGACCACTGAATGCAAACGGTACTGTCGCTGGATGCAAGGGTCGCTGTCCCAGCTCCTTTGCGTACGCTTACGACGAATCCAACGGCGGTCCCGTCTCACAGGGCTGTATCGCTGCCAAGAAAGCCGACTGTAAGTTCTTTGCGATCTGCCCGAGCTTTCTAGCTTAA

>RsTLP4 pep

MKSAVLLTCAGVVLGRTFTVYNACPYTIWPAIFTDLNVGASVPAIETGWEAPANSKRTFSVPENWAAGRIWGRTECDFSSKSGQAACLTGGCDGGLECDTKTGSGVGPVSVAEWTLGASDGHDWYDVSLVDGFNIPMRISNNVGCAIAECAVNLNQGCPDVLQGPLNANGTVAGCKGRCPSSFAYAYDESNGGPVSQGCIAAKKADCKFFAICPSFLA

> RsTLP5 CDS

ATGAAGTCCGCCGTTTTCCTCGCTTTCGTGGGCTCTGCCCTTGGTCGCACTTTTACTGTCTACAACGCGTGCCCATTTACCATCTGGCCTGCCGTCTTCACTGATCTCAATGTCGGCTCTGCTGTTCCCGCAGTTGAGACTGGCTGGGAGGCACCTGCGTGGAGTGTGAAGACCTTCACCGTTCCCGACAACTGGAAGGCCGGTCGTATCTGGGGCCGACGCAACTGCAATTTTTCGAGCAACCCCGGTCCTAACTCGTGTCTGACGGGCGGTTGCAATGGCGGTCTCCAGTGTGACTCTCGCACCGGAACCGGTGTGCCTCCTGCAAGTGTGGCGGAGTGGACTCTGAGCGCTTCCGACGGCCTCGATTGGTATGATGTGAGCTTGGTTGATGGATACAACTTGCCCATGCGCATCACCAACAACGTTGGTTGCCCGGTGGCAGATTGCGCAGTTGACCTTGGCCCAGATTGCCCCGCCCCCCTCAAGGGACCCTATGATAGCAGTGGCTTCCCCGTTGGCTGCAAGTCAGCTTGCTTTGCAAACCTTGACGGAAACCAGGGAGACTCTGGCAACTGCTGCTCCGGCTCCCATAATACCCCCGCAACCTGCCCTCCTTCCGGTGTTCAATACTATGACTACTTCAAGAGTCGCTGCCCTAACTCGTATGTCTATGCTTATGACGAGTCGAGTGGAACCGCCTTGTGGACTTGCCCTGCCTCGAAGAAGGCCGATTTTGGTTCTTCATCGGCTCAAGTCGGTATGACAACCCTGAGCAAGAATTGTGGGGTTGATTCGAGTCAGAGAAACGGATCGGGTCAAAGTTCCGAAAAAGTTGGACGGAATATTTGA

>RsTLP5 pep

MKSAVFLAFVGSALGRTFTVYNACPFTIWPAVFTDLNVGSAVPAVETGWEAPAWSVKTFTVPDNWKAGRIWGRRNCNFSSNPGPNSCLTGGCNGGLQCDSRTGTGVPPASVAEWTLSASDGLDWYDVSLVDGYNLPMRITNNVGCPVADCAVDLGPDCPAPLKGPYDSSGFPVGCKSACFANLDGNQGDSGNCCSGSHNTPATCPPSGVQYYDYFKSRCPNSYVYAYDESSGTALWTCPASKKADFGSSSAQVGMTTLSKNCGVDSSQRNGSGQSSEKVGRNI

> RsTLP6 CDS

ATGAGATCCGCTCTGTTACTGGCGTTCGTTGGCTCTGTCCTTGGTCGTACTTTCACTGTGTATAATGCGTGCCCTTTCACAATTTGGCCGGCCGTCTTTACTGACTTGAATGTGGGTTCCGCTGTTCCTGGGATTGAAACTGGCTGGGAGGCCCCGGCTTGGTCTTCGCGTACCTTTACGGTCCCAGATAATTGGAAGGCGGGGCGCATCTGGGGTCGCCGCAACTGTAACTTTTCGACGAACCCGGGCCCTAATTCATGCTTAAGTGGAGGATGCAATGGGGGGCTCCGTTGCGACTCACGAACAGGAACCGGAGTGCCACCTGCGAGCGTTGCGGAATGGACGCTTAACGCTTCTGATGGTCTGGATTGGTACGATGTAAGCCTGGTAGACGGCTACAATTTGCCTATGCGCATCACCAACAATGTCAACTGCCCCGTTGCTGATTGCCCTGTCGATCTCGGTCCCGAATGTCCCGCCCCTCTCAAAGGTCCATTCGATTCCAGTGGTTTCCCAGTTGGTTGCAAGTCTGCTTGCGCGGCCAATCTTGATGGAAATCAGGCGAACTCGAAGAACTGCTGCTCGGGTCAATACAGCACGCCTCAGACATGCCCTCCTTCGGGAGTTCAGTACTATTCCTACTTCAAGAATCGTTGCCCACGCTCGTATGTCTATGCTTACGACGAATCGAGCGGCACAGCACTATTCACTTGCCCTGCATCAAAGAAGGCTAATTTCTGA

>RsTLP6

MRSALLLAFVGSVLGRTFTVYNACPFTIWPAVFTDLNVGSAVPGIETGWEAPAWSSRTFTVPDNWKAGRIWGRRNCNFSTNPGPNSCLSGGCNGGLRCDSRTGTGVPPASVAEWTLNASDGLDWYDVSLVDGYNLPMRITNNVNCPVADCPVDLGPECPAPLKGPFDSSGFPVGCKSACAANLDGNQANSKNCCSGQYSTPQTCPPSGVQYYSYFKNRCPRSYVYAYDESSGTALFTCPASKKANF

> RsTLP7 CDS

ATGAAGTTCTCCATTGCTGCTTCTGCTCTTGCTCTCGCGAGCTCTGCTCTCGGCCGTACCTTTACCGTCTACAACGCTTGTCCCTTCACCATCTGGCCTGCTGTCTTCACTGACCTGAACGTCGGATCCGCCGTTCCTTCCATCGAGACTGGTTGGGAGGCTCCTGCCTGGAGCAAGCGCACCTTCAACGTCCCCGACAACTGGAAGGCTGGTCGTATCTGGGGCCGCCGCAACTGCAACTTTGGCTCCAACCCTGGTCCCAACTCGTGTCTCACTGGTGGCTGCAACGGGGGGCTGAGGTGCGACTCTCGCACTGGCACCGGTGTTCCTCCTGCAAGCGTCGCCGAGTGGACTTTGAGCGCCGGTGACGGCCAGGACTGGTACGATGTCTCCCTGGTCGATGGATACAACCTGCCGATGCGTATCACAAACAACGTCGGGTGCCCGGTCGCCGAGTGTGCTGTTGACCTCGGCCCGAACTGTCCTGCTCCTCTCAAGGGACCATTCGACGGATCTGGCTTCCCCGTTGGATGCAAGTCTGCTTGCGTCGCTAACTTGGACGGCAACCAAGCAAACTCGAAGAACTGCTGCTCTGGGCAGTACAGCACTCCCCAGACCTGCCCTCCTAGCGGTGTTCAGTACTACTCGTACTTCAAGAACGCATGCCCGCGCTCTTATGTCTATGCTTATGATGAGTCGAGCGGCACTGCTCTTTGGACCTGCCCCACTTCGAAGAAGGCTGACTACACCCTTACTTTCTGCCCTTGA

> RsTLP7 pep

MKFSIAASALALASSALGRTFTVYNACPFTIWPAVFTDLNVGSAVPSIETGWEAPAWSKRTFNVPDNWKAGRIWGRRNCNFGSNPGPNSCLTGGCNGGLRCDSRTGTGVPPASVAEWTLSAGDGQDWYDVSLVDGYNLPMRITNNVGCPVAECAVDLGPNCPAPLKGPFDGSGFPVGCKSACVANLDGNQANSKNCCSGQYSTPQTCPPSGVQYYSYFKNACPRSYVYAYDESSGTALWTCPTSKKADYTLTFCP

> RsTLP8 CDS

ATGGAACCTCGGGATACTTCTCGTACGCTGGTTGTATGCGTTCGGATTCATGCGAGCGAAGGGCTCTGTAAATTACATCGTAGCGAACGGGATAATATCATTCTCTGCCTTGTGGTTGCTACATCGGGAATATGTCTTCCCATTGGATTTAGCATGCTGGTACTTCACTTCGGGTACCAGTATGCTCTGCTCGAGTCGTTCGTCGTCGGCGCGGCGCTTAGCGCGACATCCATGAATGTCTATCTGCACCAGCGGGATGGCCGAGATGTCCGACGGTTGCATGTGCTTGCACATCACTTCACTCCTGCCGATGCGCTTGACCGACGCTTAGCCCAACCGGGCTCACGTGCGTCTTGTGGACATGATGAGAATGCTGGATTTGCGCGACCTGGCTGGGCTGGGACGACAAGCATCATCGTAGAAAGTTCTACTCATCCACCCCTACAGCTTCTCTCAAGTTCGACTCAACAAACATGAAGTTCATCATCGCTGCTTCTGCTCTTGCTCTTGCGAGCTCTGCTCTCGGCCGTACCTTTACCGTCTACAACGCTTGCCCCTTCACCATCTGGCCTGCTGTCTTTACTGACCTGAACGTCGGATCCGCCGTTCCTTCCATCGAGACTGGTTGGGAGGCTCCTGCCTGGAGCAAGCGCACCTTCAACGTCCCCGACAACTGGAAGGCTGGTCGTATCTGGGGTCGCCGCAACTGCAACTTCGGCTCCAACCCTGGTCCCAACTCGTGTCTCACTGGTGGCTGCAACGGGGGGCTGAGGTGCGACTCTCGCACTGGCACTGGTGTTCCTCCTGCAAGCGTCGCCGAGTGGACTTTGAGCGCCGGTGACGGCCAGGACTGGTACGATGTCTCCCTGGTCGATGGATACAACCTGCCGATGCGTATCACAAACAACGTCGGGTGCCCGGTCGCCGAGTGTGCTGTTGACCTCGGCCCGAACTGTCCTGCTCCTCTCAAGGGACCGTTCGACGGATCTGGCTTCCCCGTTGGATGCAAGTCTGCTTGCGTCGCTAACTTGGACGGCAACCAAGCAAACTCGAAGAACTGCTGCTCTGGGCAGTACAGCACTCCCCAGACCTGCCCTCCTAGCGGTGTTCAGTACTACTCGTACTTCAAGAACGCATGCCCGCGCTCGTACGTCTATGCTTATGACGAGTCGAGCGGCACTGCTCTTTGGACCTGCCCCACTTCGAAGAAGGCTGACTGTGTTGTCTTGAACTATTCCGTGTCTGTGAATGACCTTCGAAAGTGTCCTCGGTCATGTGTCCGAGAAGCCGGGCTCGGTGCGGGCCAAGGAATACCCGGCCACATGATTTCAGCGCTGAGATAA

>RsTLP8 pep

MEPRDTSRTLVVCVRIHASEGLCKLHRSERDNIILCLVVATSGICLPIGFSMLVLHFGYQYALLESFVVGAALSATSMNVYLHQRDGRDVRRLHVLAHHFTPADALDRRLAQPGSRASCGHDENAGFAQTWLGWDDKHHRRKFYSSTPTASLKFDSTNMKFIIAASALALASSALGRTFTVYNACPFTIWPAVFTDLNVGSAVPSIETGWEAPAWSKRTFNVPDNWKAGRIWGRRNCNFGSNPGPNSCLTGGCNGGLRCDSRTGTGVPPASVAEWTLSAGDGQDWYDVSLVDGYNLPMRITNNVGCPVAECAVDLGPNCPAPLKGPFDGSGFPVGCKSACVANLDGNQANSKNCCSGQYSTPQTCPPSGVQYYSYFKNACPRSYVYAYDESSGTALWTCPTSKKADCVVLNYSVSVNDLRKCPRSCVREAGLGAGQGIPGHMISALR

>RsTLP9 CDS

ATGAAGTCCGCCGCTCTCCTCGCTTTCGCCGGTGCTGCTCTCGGCCGTACGTTCACCGTTTACAACGCGTGTCCGTTCACCATCTGGCCTGCAGTTTTCACCGATCTCAACGTCGGTTCGGCAGTTCCTTCGATCGAGACTGGCTGGGAAGCTGCGGCTTACACTAAGCGCACCTTCACCGTTCCTGACAACTGGAAGGCCGGTCGCATCTGGGGTCGTCGCAACTGCAACTTTGCGAGCAACCCTGGCCCCAACTCTTGCTTGACCGGTGGCTGCAACGGTGGTCTCAAGTGCGACTCTCGTACCGGTACTGGTGTTCCCCCAGCTAGTGTTGCCGAGTGGACCTTGAGCGCTTCGGATGGTCTCGATTGGTATGATGTGAGCTTGGTCGATGGGTACAACCTGCCCATGCGTATCTCCAACAACGTGGGCTGTGAAGTTGCTGAATGCGCTGTTGACCTTGGCCCCAACTGCCCTGCTCCTCTCAAGGGGCCCTTCGACAGCAGCGGTTTCCCCGTTGGCTGCAAGTCTGCTTGCGGTGCCAACCTCGACGGAAACCAGGCCAACTCTGCTAACTGTTGCTCTGGCTCCCACAACACCCCTGCGACCTGCCCTCCGTCTGGTGTTCAGTACTACTCCTACTTCAAGAACGCTTGCCCCCGCTCCTACGTCTATGCTTATGACGAGTCCAGCGGCACCGCTTTGTGGACATGCCCTGCTTCGAAGAAGGCTGACTACACTCTTACTTTCTGCCCGTGA

>RsTLP9

MKSAALLAFAGAALGRTFTVYNACPFTIWPAVFTDLNVGSAVPSIETGWEAAAYTKRTFTVPDNWKAGRIWGRRNCNFASNPGPNSCLTGGCNGGLKCDSRTGTGVPPASVAEWTLSASDGLDWYDVSLVDGYNLPMRISNNVGCEVAECAVDLGPNCPAPLKGPFDSSGFPVGCKSACGANLDGNQANSANCCSGSHNTPATCPPSGVQYYSYFKNACPRSYVYAYDESSGTALWTCPASKKADYTLTFCP

> RsTLP10 CDS

ATGAAGTCCGCCGCTCTCCTCGCTTTCGCCGGTGCTGCTCTCGGCCGTACGTTCACCGTTTACAACGCGTGCCCGTTCACCATCTGGCCTGCAGTTTTCACCGATCTCAACGTCGGTTCGGCAGTTCCTTCGATCGAGACTGGCTGGGAAGCTGCGGCTTACACTAAGCGCACCTTCACCGTTCCTGATAACTGGAAGGCCGGTCGCATCTGGGGTCGTCGCAACTGCAACTTTGCGAGCAACCCTGGCCCCAACTCTTGCTTGACCGGTGGCTGCAACGGTGGTCTCAAGTGCGACTCTCGTACCGGTACTGGTGTTCCCCCGGCTAGTGTTGCCGAGTGGACCTTGAGCGCTTCGGATGGTCTCGATTGGTATGATGTGAGCTTGGTCGATGGGTACAACCTGCCCATGCGTATCTCCAACAACGTTGGCTGTGAAGTTGCTGAATGCGCTGTTGACCTTGGCCCCAACTGCCCTGCTCCTCTCAAGGGGCCCTTCGACAGCAGCGGTTTCCCCGTTGGCTGCAAGTCTGCTTGCGGTGCCAACCTCGACGGAAACCAGGCCAACTCTGCTAACTGCTGCTCTGGCTCCCACAACACCCCTGCGACCTGCCCTCCGTCTGGTGTTCAGTACTACTCCTACTTCAAGAACGCTTGCCCCCGCTCCTACGTCTATGCTTATGACGAGTCCAGCGGCACCGCTTTGTGGACATGCCCTGCTTCGAAGAAGGCTGACTACACTCTCACTTTCTGCCCGTGA

>RsTLP10 Pep

MKSAALLAFAGAALGRTFTVYNACPFTIWPAVFTDLNVGSAVPSIETGWEAAAYTKRTFTVPDNWKAGRIWGRRNCNFASNPGPNSCLTGGCNGGLKCDSRTGTGVPPASVAEWTLSASDGLDWYDVSLVDGYNLPMRISNNVGCEVAECAVDLGPNCPAPLKGPFDSSGFPVGCKSACGANLDGNQANSANCCSGSHNTPATCPPSGVQYYSYFKNACPRSYVYAYDESSGTALWTCPASKKADYTLTFCP
